# Modern Treatment of Masculine Gonorrhea

**Published:** 1907-07

**Authors:** 


### Modern Treatment of Masculine Gonorrhea.

Stock, of Cologne, reviewing the modern treatment of masculine gonorrhea (*Medico,* 1907, No. 7), emphasises the importance of internal medication. A purely local treatment is often unsatisfactory and cannot be used continuously when complications are present. Of the internal remedies he prefers arhovin because, though vigorously antigonorrheaic, it is free from the untoward effects of the balsams. Arhovin internally alone is indicated in acute anterior urethritis with intense inflammation, pain on urination and swelling of the meatus. It is also a complete substitute for specific medication when complications have arisen, such as epididymitis, cystitis, inflammation of the urinary tract. In chronic gonorrhea it is sometimes astonishingly effective.

Zorn of Bruenn also lauds arhovin as our very best internal antigonorrheiac and details a number of cases of anterior and posterior urethritis, acute urethrocystitis and chronic gonorrheal cystitis in which strikingly convincing results were obtained (*Fortschritte d. Medicin,* 1906, No. 34). In vulvovaginitis he also employed arhovin bougies and in fluor albus gonorrhoica arhovin globules, with much satisfaction. In fluor albus the course of the disease was not shortened visibly, but the analgetic effect of the arhovin was gratifying.

